# Nutrition Therapy, Glucose Control, and Brain Metabolism in Traumatic Brain Injury: A Multimodal Monitoring Approach

**DOI:** 10.3389/fnins.2020.00190

**Published:** 2020-03-24

**Authors:** Pedro Kurtz, Eduardo E. M. Rocha

**Affiliations:** ^1^Department of Neurointensive Care, Instituto Estadual do Cérebro Paulo Niemeyer, Rio de Janeiro, Brazil; ^2^Department of Intensive Care Medicine, Hospital Copa Star, Rio de Janeiro, Brazil

**Keywords:** nutrition therapy, cerebral microdialysis, glucose control, brain glucose, neurointensive care

## Abstract

The goal of neurocritical care in patients with traumatic brain injury (TBI) is to prevent secondary brain damage. Pathophysiological mechanisms lead to loss of body mass, negative nitrogen balance, dysglycemia, and cerebral metabolic dysfunction. All of these complications have been shown to impact outcomes. Therapeutic options are available that prevent or mitigate their negative impact. Nutrition therapy, glucose control, and multimodality monitoring with cerebral microdialysis (CMD) can be applied as an integrated approach to optimize systemic immune and organ function as well as adequate substrate delivery to the brain. CMD allows real-time bedside monitoring of aspects of brain energy metabolism, by measuring specific metabolites in the extracellular fluid of brain tissue. Sequential monitoring of brain glucose and lactate/pyruvate ratio may reveal pathologic processes that lead to imbalances in supply and demand. Early recognition of these patterns may help individualize cerebral perfusion targets and systemic glucose control following TBI. In this direction, recent consensus statements have provided guidelines and recommendations for CMD applications in neurocritical care. In this review, we summarize data from clinical research on patients with severe TBI focused on a multimodal approach to evaluate aspects of nutrition therapy, such as timing and route; aspects of systemic glucose management, such as intensive vs. moderate control; and finally, aspects of cerebral metabolism. Research and clinical applications of CMD to better understand the interplay between substrate supply, glycemic variations, insulin therapy, and their effects on the brain metabolic profile were also reviewed. Novel mechanistic hypotheses in the interpretation of brain biomarkers were also discussed. Finally, we offer an integrated approach that includes nutritional and brain metabolic monitoring to manage severe TBI patients.

## Introduction

Patients with traumatic brain injury (TBI) who overcome the initial injury commonly sustain severe metabolic and physiological alterations, which greatly enhance the consumption of the body mass. However, before the 1980s, the practice of nutrition therapy was to start feeding only when normal gastrointestinal function recovered or when the capacity of the patient to eat was adequately present (Twyman, 1997; Bistrian et al., 2011). The main consequences of traumatic injury in body composition are weight loss; consumption of lean body mass, mainly skeletal muscle mass; negative nitrogen balance; and water and salt retention. These basic alterations will leave these patients prone to immune depression and increased susceptibility to infection, sepsis, and generalized organ failure, leading to prolonged intensive care unit (ICU) and hospital stays and increased morbidity and mortality. Following the initial insult, a state of hypermetabolism together with hypercatabolism will ensue, mainly due to hypersecretion of endogenous catabolic hormones such as corticosteroids, catecholamines, and glucagon, in association with interleukins IL-1, IL-6, and tumor necrosis factor-α (TNF-α). Acute secondary events post-TBI also lead to widespread changes in neurotransmitters (Twyman, 1997; Bistrian et al., 2011; Erdman et al., 2011).

## Nutrition Therapy

### Timing of Initiation

In the presence of an acute catabolic state and other common pathophysiologic features of severe brain injury, it is mandatory to avoid delays in starting nutrition therapy to preserve, as much as possible, the skeletal muscle mass, vital organ function, and cerebral metabolic homeostasis. Following this important corollary, nutrition therapy should be initiated early, ideally within the first 24 h after injury, and provide more than 50% of resting energy expenditure (REE) with 1.0 to 1.5 g protein/kg, for the 2 weeks subsequent to the injury (Perel et al., 2006; Bistrian et al., 2011). This intervention is critical in limiting the intensity of the inflammatory response to TBI and improving the outcome. Hart and collaborators showed that patients who were not fed within 5 to 7 days after TBI had a 2- to 4-fold increase in the likelihood of death, respectively. Consequently, the authors stated that nutrition is a significant predictor of mortality after TBI, and together with the prevention of arterial hypotension, hypoxia, and intracranial hypertension, is one of the few therapeutic interventions that can directly affect TBI outcome (Hartl et al., 2008).

In a systematic review including 13 randomized controlled trials and three non-randomized prospective studies on nutritional support for TBI patients, Wang and colleagues demonstrated the beneficial effects of early nutrition on reducing mortality, improving functional outcomes, and decreasing infectious complications. Their results also lent support to the use of small bowel feeding and immune-enhancing formulae to reduce infectious complications in this clinical situation (Wang et al., 2013).

### Route of Access: Digestive Tract (Enteral Nutrition) Versus Intravenous (Parenteral Nutrition)

Justo Meirelles and Aguillar-Nascimento investigated whether early enteral nutrition (EN) or parenteral nutrition (PN) would differ in energy and protein supply and serum glucose levels in the acute phase response in patients with TBI. Twenty-two patients with moderate TBI [Glasgow Coma Scale 9–12 (GCS)] were randomized to receive isocaloric and isonitrogenous EN (*n* = 12) and PN (*n* = 10). The authors recorded the daily quantities of calories and nitrogen (N) administered, the nitrogen balance, and the daily serum levels of glucose, C-reactive protein (CRP), and albumin for 5 consecutive days. They also compared the length of stay and mortality as clinical endpoints. Both groups showed significant progressive caloric deficits (*p* = 0.001) but without any difference between them. The average serum glucose level was higher in the PN group than in the EN group, 134.4 vs. 113.2 mg/dL, *p* < 0.001, respectively. A trend (*p* = 0.06) was observed in the 24 h urinary N loss that was greater in the PN group, which received significantly greater amounts of N than the EN group (*p* < 0.05), but the N balance was similar in both groups. The serum levels of CRP increased; however, those of albumin did not change, and the mortality was 9.1% (twovcases, one in each group). The authors concluded that both routes were able to provide an increasing number of calories, with a cumulative mean of 6300 kcal for the period studied, but with progressive caloric deficits that were not stated. Another important observation was that TBI patients lose increased quantities of N, independent of the route of nutrition therapy, leading to increased N requirements. In comparing PN to EN, the former leads to greater hyperglycemia, but there was no influence of the route in both the early acute phase inflammatory response or the clinical outcomes (Justo Meirelles and de Aguilar-Nascimento, 2011).

Furthermore, TBI patients usually receive either osmotic diuretics or hyperosmolar fluids, such as hypertonic saline, for the treatment of increased intracranial pressure (ICP). This adds to the challenge of clinicians to prevent increased fluid and electrolyte derangements, which are also common after neurologic injury. Thus, nutrition therapies should consider precise fluid resuscitation strategies, with special attention to strict electrolyte monitoring, specific to TBI patients, with the purpose of avoiding excessive fluid, electrolyte, and glucose shifts that could be detrimental to these patients (Rhoney and Parker, 2006; Cook et al., 2008; van der Jagt, 2016).

The recently updated Guidelines for the Management of Severe TBI from the Brain Trauma Foundation state that early transgastric jejunal feeding is recommended to reduce the incidence of ventilator-associated pneumonia (Carney et al., 2017). Corroborating this recommendation, Chiang and collaborators reported data from the medical records of TBI patients with GCS scores of 4 to 8 admitted to 18 hospitals in Taiwan, excluding those with GCS ≤ 3. They included 145 EN patients receiving appropriate kcal and nutrients 48 h post-trauma and compared them with 152 non-EN controls matched for sex, age, body weight, initial GCS score, and operative status^11^. The EN patients showed a greater survival rate and GCS score on the 7th day in the ICU and better outcomes at 1-month post-injury. The hazard ratio for the non-EN patients after adjusting for age, sex, initial GCS score, and recruitment period was 14.63 (95% CI 8.58–24.91) compared with EN patients. Additionally, the comparison of the GCS scores between these two groups of patients showed significant clinical improvement during the first 7 ICU days for the EN patients. These results confirm that EN, within 48 h post-injury, is associated with better survival, GCS recovery, and the functional outcome of TBI patients, especially those with a GCS score of 6–8 (Chiang et al., 2012).

The advantages and disadvantages of parenteral and enteral nutrition are well-documented and defined. Classically, PN is frequently associated with higher rates of infection, immunosuppression, hyperglycemia, hepatic steatosis, diminished gastrointestinal (GI) integrity, and the expression of gut -associated lymphoid tissue (GALT). On the other hand, EN stimulates GI post-prandial hyperemia, enhancing mucosal blood flow, which counterbalances the alterations in GI blood flow due to situations of increased intrathoracic pressure during vasopressor use, leading to an increase in GALT expression. Furthermore, EN provides a better quality of macro- and micronutrients such as medium-chain triglycerides and fiber, leading to the production of short-chain fatty acids (Cook et al., 2008; Bistrian et al., 2011; Wang et al., 2013). Based on this rationale and the data available, we agree that early EN is the best approach to nutrition therapy in patients with severe TBI.

### Standard or Immune-Enhancing Enteral Nutrition

Following acute neurotrauma, a phase of neuroinflammation, free radical generation, excitatory toxicity, and oxidative stress ensues. Since the early administration of enteral feeding to patients with TBI is reasonably well-established, the combination of immune-modulating nutrient supplementation, also well-known to be an adjuvant in maintaining and supporting the structural integrity of the gastrointestinal mucosa and its immunological function, should greatly benefit patients suffering from TBI in their return to near-normal cerebral homeostasis. The general principles of the immune-enhancing strategy focus on the supplementation of a combination of immune-modulating nutrients – glutamine, arginine, omega-3 fatty acids, and nucleotides – with the aim of promoting brain tissue recovery and minimizing neuronal loss in the vulnerable, but still viable, areas surrounding the primary brain injury (Rai et al., 2017).

Rai and associates (Rai et al., 2017) recently evaluated the effect of an immunonutrition formulation on biomarkers – IL-6, glutathione, CRP, total protein, and albumin in 36 patients with moderate-to-severe head injury (GCS 3–12) admitted to a neurosurgical ICU over a 6-month period and who required EN. The patients were divided into groups A (Immune-enhancing EN diet) and B (Standard EN diet) and received an isocaloric [1.4 x basal energy expenditure (BEE)] or isonitrogenous formulation, respectively, with each group, starting EN 24 to 48 h after admission or surgery. By day 5, EN patients in group A showed a significant decrease in IL-6 (*p* < 0.001) and a significant increase in glutathione (*p* < 0.049) serum levels, as well as an increased total protein level at the end of the study (*p* = 0.016). Moreover, there was a trend toward higher serum levels of albumin and lower serum levels of CRP in group A (*p* = NS). In conclusion, the authors stated that immunonutrition significantly decreased inflammation, lowering IL-6 serum levels and increasing antioxidant defense through the endogenous increase in glutathione levels and enhanced visceral nutritional status, with an increase in total visceral proteins (Rai et al., 2017). In a similar study, Khorana and associates (Chiang et al., 2012) randomized 40 moderate-to-severe TBI patients either to an immune-enhancing or a standard diet delivered intragastrically. Again, the levels of IL-6 were markedly reduced on day 3 (*p* = 0.002) (IL-10 did not change) in the immune-enhancing diet group compared to the standard diet group. Thus, both studies demonstrate that short-term enteral feeding in the post-traumatic phase of acute brain injury can reduce cytokine levels, suggesting that systemic inflammatory response syndrome (SIRS) might be modulated by this type of feeding (Khorana et al., 2009).

More recently, based on the fundamental concepts of immunonutrition, Painter and associates conducted a retrospective analysis of patients with isolated TBI admitted to a level one urban trauma center (Painter et al., 2015). A total of 240 patients, severely brain injured upon admission, defined as a head abbreviated-injury severity (AIS) score of at least 3 and/or GCS ≤ 8, with computed tomography evidence of head injury, met inclusion criteria for the analysis. Of these, 114 received the standard formula, and 126 received the immune- enhancing formula (IEN). Both groups were similar in terms of age, initial hospital GCS score, injury-severity score (ISS), and AIS scores. The two groups had similar durations of tube feeding and similar initial pre-albumin levels. In subsequent weeks 2 and 3, these levels were significantly higher in the IEN group and remained higher, although not significantly, at weeks 4 and 5. In addition, IEN patients had less bacteremia during hospitalization (10.3 vs. 19.3%, *p* < 0.05) but similar rates of urinary tract infection (16.7 vs. 20.2%, *p* = 0.48), pneumonia (57.9 vs. 57.0%, *p* = 0.89) and *Clostridium difficile* infection (4.0 vs. 5.3%, *p* = 0.63). Interestingly, the IEN group had significantly more mechanical ventilation days and longer ICU length of stay (LOS, *p* ≤ 0.02) but showed no difference in hospital LOS and mortality (7.5 vs. 9.6%, *p* = 0.88). In conclusion, severe acute TBI patients who received IEN were more likely to have higher prealbumin levels, reflecting improved nutritional status during hospitalization, and they developed less nosocomial infections, namely, bacteremia (Painter et al., 2015).

### Estimation or Measurement of Energy Requirements

Some decades ago, Clifton and associates studied 57 patients with TBI and demonstrated, after the analysis of 312 energy expenditure measurements, that patients in coma secondary to head injury present a hypermetabolic state with hypercatabolism of whole-body protein, leading to a highly negative nitrogen balance, similar to patients with severe multiple trauma or extensive burns (Clifton et al., 1986). However, the intensity of this response can vary widely among comatose head-injured patients and may be influenced by factors such as temperature, GCS score, muscle activity and tone, and time of measurement after the primary injury. The measured resting energy expenditure (mREE) after TBI varied from 100 to 125% of expected values if patients were paralyzed or in a barbiturate coma. This value increases to > 125% up to 250% following localizing and posturing after stimulation, increasing muscle tone, sweating, fever, and increasing the number of days after the primary trauma (Clifton et al., 1986).

Apparently, contradicting these findings, Osuka and associates reported on 10 adult patients, and Mtwaeh and associates studied 13 children (≤ 18 years of age and ≥ 10 kg of body weight) with severe TBI who were mechanically ventilated under controlled normothermia with sedation and neuromuscular blockade (Osuka et al., 2013; Mtaweh et al., 2014). Both studies showed lower than expected mREE – 87.2 ± 10 and 70.2 ± 3.8%, respectively – in comparison to their BEE predicted by the Harris-Benedict equation. Therefore, it is evident that TBI itself causes an intrinsic increase in metabolism, which would lead to an equivalent increase in caloric support. However, similar to previous studies, most TBI patients were on mechanical ventilation, under controlled normothermia, under deep sedation, and sometimes with a neuromuscular blockade. Altogether, the data suggest that despite an intrinsic increase in metabolism in TBI patients, therapeutic measures in the Neuro ICU may partially reduce this response (Clifton et al., 1986; Osuka et al., 2013; Mtaweh et al., 2014).

Thus, indirect calorimetry is the gold standard by which to measure REE in critically ill patients, and the clinical application of mREE to target nutritional requirements or monitor nutritional support is already established. In summary, despite the hypermetabolic state triggered by TBI observed in day-to-day clinical practice, this acute metabolic state is reduced by current routine neurocritical care measures. Based on the diversity of the data on this specific matter, the Committee on Nutrition, Trauma, and the Brain Food and Nutrition Board of the Institute of Medicine suggests that permissive underfeeding (initially 50% of energy needs, progressing up to 25–30 kcal/kg/day in the first 2 weeks) is probably an appropriate feeding strategy to be initiated within the first 24 h. Moreover, the Brain Trauma Foundation proposes feeding TBI patients to attain basal caloric replacement at least by the 5th day and at most, by the 7th day post-injury to decrease mortality (Badjatia and Vespa, 2014; Carney et al., 2017).

### Macronutrients

A corollary of REE determination in this clinical setting is whether carbohydrates or fats function as the best source of energy substrate to be offered. The metabolic response to trauma, in general, is the classical hypersecretion of the hormonal triad of stress injury, such as glucocorticoids, catecholamines, and glucagon. Concomitantly, TBI *per se* causes an intrinsic increase in metabolism, derived from a still unknown centrally mediated mechanism, clearly demanding more caloric and protein administration. Consequently, this metabolic environment stimulates the increase in glycogenolysis and gluconeogenesis, the latter mainly from tricarbonated substrate derivatives (lactate, pyruvate, and alanine), without a prompt parallel enhancement in insulin secretion and sensitivity, leading to acute hyperglycemia (Twyman, 1997; van der Jagt, 2016). A number of studies in general ICU patients demonstrated that the control of this hyperglycemic response using exogenous insulin, can lead to significant improvements in outcomes of critically ill patients. On the other hand, the use of intensive insulin therapy with tight glycemic control could have overall deleterious effects for the patients, especially for those with severe TBI, possibly due to hypoglycemia (Investigators et al., 2009; Blackburn et al., 2010; Nice-Sugar Study Investigators for the Australian, New Zealand Intensive Care Society Clinical Trials Group, and the Canadian Critical Care Trials Group et al., 2015; van der Jagt, 2016). The ideal target of glucose control remains controversial in patients with TBI. Although most authors agree that hypo- and hyperglycemia should be avoided, the exact thresholds are still undefined.

**FIGURE 1:**
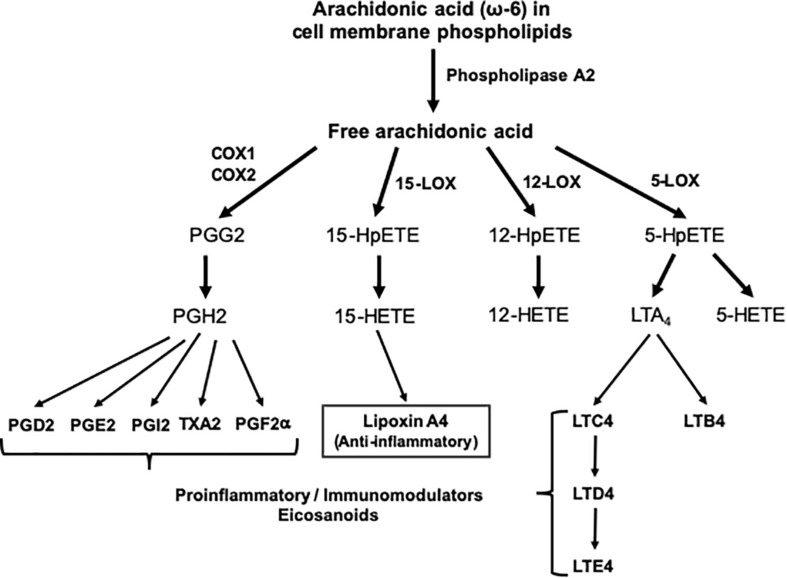
Outline of the pathway of eicosanoid synthesis from arachidonic acid. COX, cyclooxygenase; HETE, hydroxieicosatetraenoic; HpETE, hydroxiperoxyeicosatetraenoic; LOX, lipoxygenase; LT, leukotriene; PG, prostaglandin; TX, thromboxane.

**FIGURE 2:**
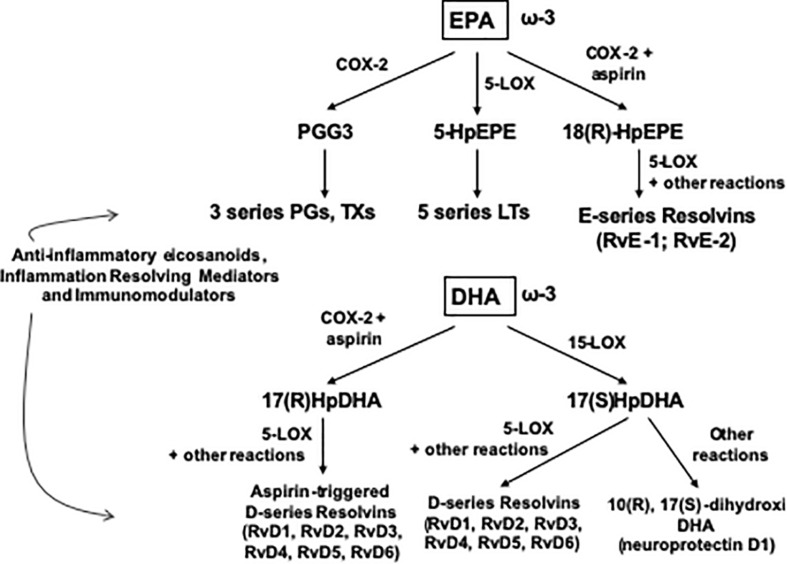
Outline of the pathway of synthesis of Resolvins and related mediators from EPA and DHA. COX, cyclooxygenase; HpDHA, hydroxiperoxidocosahexaenoic; HpEPE, hydroxiperoxyeicosapentaenoic; LOX, lipoxygenase; LT, leucotriene; PG, prostaglandin; Rv, resolvin, TX, tromboxane.

The skeletal muscle can metabolize and clear exogenous lipids administered to trauma patients through its oxidation as an energy substrate. The complete oxidation of fatty acids includes three stages: (1) β oxidation, (2) Citric acid cycle, and (3) Oxidative phosphorylation. In this metabolic pathway, triacylglycerols produce more than half of the energy utilized by the liver, heart and skeletal muscles. Additionally, the type of lipid to be offered in TBI is important since omega-6 fatty acids, mainly arachidonic acid derived from linoleic acid, modulate innate immunity and are inflammatory (series 2 and 4 eicosanoids), while omega-3 fatty acids (series 3 and 5 eicosanoids) can counterbalance the inflammatory effects of omega-6 fatty acids and favorably modulate the innate immunity involved in inflammation. Thus, the n-3 polyunsaturated fatty acids (PUFAs), eicosapentaenoic (EPA) and docosahexaenoic (DHA), when released from the plasma membrane phospholipids in the process of TBI are converted into resolvins and protectins (e.g., neuroprotectin D1), which actively promote resolution of inflammatory processes by downregulating the NF-κB pathway and promoting the clearance of neutrophils, activating synaptic plasticity and cytoskeletal assembly, respectively (Walike, 1969) (see Figures 1, 2).

Consequently, Hasadsri and colleagues stated that n-3 PUFAs mitigate the consequences of several key pathological pathways in TBI, such as mitochondrial malfunction, apoptotic cell death, glutamate-triggered excitotoxicity, and injury-induced oxidative stress and inflammation. In summary, n-3 PUFAs may therefore play a critical role in the restoration of cellular energetics and the repair of neuronal damage after TBI (Hasadsri et al., 2013).

Throughout the process of human evolution, the diet was composed of approximately equal amounts of n-6 and n-3 PUFAs (ratio of 1-2:1, respectively); however, in contrast, the present-day Western diet is very high in n-6 PUFAs and relatively deficient in n-3 PUFAs (ratio of 10-20:1, respectively). Then, the current recommendations for lipids in the event of trauma, including TBI, suggest 25 to 40% of the total calories, with a ratio of n-3/n-6 PUFAs ranging from 2:1 to 8:1 and a concentration from 2 to 6 g/day of n-3 PUFAs (van der Meij et al., 2011; Scrimgeour and Condlin, 2014).

The complex neuro-endocrine-immune response to TBI triggers the enhancement of gluconeogenesis secondary to skeletal muscle protein hypercatabolism, mainly through the activation of the ubiquitin-proteasome system. This exacerbated body protein catabolism for endogenous calories can be attenuated by the provision of energy from 100 to 150% of REE and can be further decreased by providing high levels of dietary protein (Clifton et al., 1986; Frankenfield, 2006; Cook et al., 2008; Charrueau et al., 2009; Frankenfield et al., 2009).

Several authors have shown that TBI patients lose increased amounts of nitrogen independently of the corresponding administered quantity and that nitrogen excretion increases concomitantly and steadily for up to 4 weeks from the primary injury, making it very difficult to equalize the nitrogen balance. Clifton and colleagues, corroborated by several others, stated that the attempt to block nitrogen wasting after TBI, by increasing protein administration, has resulted in further elevation of protein catabolism so that only 50% of the nitrogen supply has been retained at high levels of protein intake (Clifton et al., 1986; Frankenfield, 2006; Cook et al., 2008; Charrueau et al., 2009; Frankenfield et al., 2009; Justo Meirelles and de Aguilar-Nascimento, 2011).

Thus, clinical guidelines from specialty societies recommend an early provision of 1.5 g to 2.0 g/kg/day of protein, accompanied by at least 50% of energy needs up to 25–30 kcal/kg/day, which is likely appropriate for both TBI and any associated critical injury. A corollary of this concept of programmed short permissive underfeeding is the interrelationship of energy and protein intake. When energy intakes are limited, supplying greater levels of protein, up to 1.5 g/kg/day, will improve the preservation of fat-free mass and improve protein synthetic rates (McClave et al., 2016). The immune-modulating diets could also be an appropriate option for fulfilling this gap of protein supply for TBI patients, aiming at the attenuation of exacerbated negative nitrogen balance (Charrueau et al., 2009; Painter et al., 2015; McClave et al., 2016; Rai et al., 2017).

### Hyperosmolar Therapy and Metabolic Alterations

Patients with acute TBI are frequently exposed to hyperosmolar fluids, such as hypertonic saline, which has gained significant interest in current neurocritical therapy because they are devoid of dehydrating properties and have several other beneficial advantages, such as increasing cardiac output, decreasing peripheral vascular resistance and afterload, increasing anti-inflammatory properties and increasing intravascular volume (Rhoney and Parker, 2006; Cook et al., 2008; van der Jagt, 2016). Therefore, clinicians must be aware of the quantities and concentrations of protein and sodium being administered for patients on EN because the intake of large amounts of protein and insufficient water, with a moderate intake of sodium, may constitute the main cause of Tube Feeding Syndrome (Hypernatremia, Azotemia, and Dehydration) (Gault et al., 1968; Walike, 1969).

Since TBI patients are frequently hypercatabolic due to the nature of their primary insult, the constantly increased protein catabolism results in the presentation of an enhanced nitrogen load for excretion by the kidneys, which demands a greater urine volume. Thus, the basic pathophysiologic event in this situation is the excessive osmotic load presented to the kidneys and its incapacity to dilute the excess nitrogen, together with other electrolytes, for excretion (healthy subjects concentrate up to 1300 mOsm/kg/day of solute load).

Effectively, patients with TBI progressing through the acute and subacute phases of injury, presenting hypermetabolism and exacerbated whole body protein catabolism, under osmotic therapy (mannitol 20% or hypertonic saline 2–3%) to decrease ICP and cerebral edema and being fed with high-protein EN, are prone to developing hypernatremia. On the other hand, hypernatremia is a frequent component of the treatment of cerebral edema and could be classified as suggested in Table 1. Thus, clinicians must be aware of the current status of natremia and serum osmolarity of TBI patients to adequately modulate their nutrition therapy to avoid hypernatremia and hyperosmolarity, aiming for a decrease in morbidity and consequently, the mortality of these patients.

**TABLE 1:** Hypernatremia severity (Adrogue and Madias, 2000; Aiyagari et al., 2006; Kolmodin et al., 2013).

Type	Maximum serum sodium (mEq/L)
(a) Mild	≥151 ≤ 155
(b) Moderate	≥156 ≤ 160
(c) Severe	>160

### Nutrition Monitoring, Prevention of Complications, and Suggested Protocol for Nutrition Therapy Implementation

In the process of feeding patients with TBI, one has to consider the different phases of illness, namely: (a) Acute, or Resuscitation; (b) Persistent Inflammation, Hyperflux, Support or Maintenance; (c) Chronic disease, or Prolonged Critical Illness; and (d) Flux, or Rehabilitation (Pamplin et al., 2011). To implement the most appropriate feeding regimen, the clinician should aim at monitoring and maintaining body mass consumption, preserving adequate brain metabolism and homeostasis, and avoiding specific complications related to each different phase. Table 2 summarizes the main metabolic and clinical situations to be monitored and/or avoided. Moreover, as shown by the scientific evidence above and in order to organize a nutrition therapy protocol for patients with TBI, aiming at its adequate and safe application in daily clinical practice, the summarized recommendations for nutrition therapy implementation provided in Table 3 should be followed.

**TABLE 2:** Monitoring parameters of nutrition therapy (Citerio et al., 2015; McClave et al., 2016).

Metabolic/clinical parameter	Clinical/laboratory marker	Prophylaxis/treatment or monitoring
Glycemia	Hyper or hypoglycemia	Insulin/adequate diet Avoid intensive insulin therapy (Citerio et al., 2015) Risk of hypoglycemia
Somatic/visceral protein	Total urinary nitrogen Serum total protein, transferrin, transthyretin	Adequate protein delivery (McClave et al., 2016) Negative nitrogen balance
Serum hyperosmolarity	Hypernatremia ≥ 160 mEq/L Chloremia ≥ 110 mEq/L	Balanced solutions or Free water volume delivery (avoid 5% dextrose solution)
Serum hypo-osmolarity	Hyponatremia ≤ 135 mEq/L	Hyperosmolar/hypertonic Solution administration
Refeeding syndrome	Hypophosphatemia, hypokalemia, and hypomagnesemia	Slow diet administration (McClave et al., 2016; Carney et al., 2017) Add phosphate, potassium, and magnesium if needed
Overfeeding	REE measurement	Perform indirect calorimetry (Le Roux et al., 2014) Evaluate clinical symptoms
Underfeeding	REE measurements Bioimpedance analysis Serum visceral proteins	Adjust for adequate caloric and protein Administration (McClave et al., 2016) Evaluate nutrition type/quality
Diarrhea	Excessive stool frequency and loose consistency	Avoid GI prokinetic agents Change diet type – add fiber Test for *Clostridium difficile*
Abdominal distention	Decreased bowel sounds Bloated and tense abdomen	Measure gastric residuals (Le Roux et al., 2014) Start GI prokinetic agents Initiate jejunal feeding
Gastric paresis Gastric residuals	Complication: aspiration pneumonitis	Perform plain chest X-ray Measure GI enzymes in tracheal aspirate Head of bed ≥ 30° ≤ 45° GI prokinetics/jejunal feeding (McClave et al., 2016)

*REE, resting energy expenditure; GI, gastrointestinal.*

**TABLE 3:** Recommendations for nutrition therapy (Citerio et al., 2015; McClave et al., 2016; Carney et al., 2017; Singer et al., 2019).

Nutrition issue	Parameter	Comments/clinical implications
Route for access	Preferred: Enteral (gastric, jejunal – transpyloric)	Better GI integrity
		GALT stimulation
		Gastric: not recommended – formation of residuals
		Jejunal transpyloric: preferred
	Parenteral	May worsen hyperglycemia
		Immunosuppressive
Timing to initiate	Early: 24–48 h of admission	Improved survival
Position	Post-pyloric Ligament of treitz and over	3^rd^ portion of duodenum
		Optimal placement
		Best GI tolerance
Type	Enteral nutrition: standard vs. immune enhancing	Inferior nutritional/inflammatory/immune responses
		Better visceral proteins
		Better modulation of inflammatory response
		Lower rates of infection
REE determination	Formula estimation vs. indirect calorimetry (IC)	Best equations:
		Penn State 2006 (Frankenfield, 2006; Frankenfield et al., 2009)
		Swinamer 1990 (Swinamer et al., 1990)
		Ireton-Jones 1992 (Ireton-Jones et al., 1992; Ireton-Jones and Jones, 2002)
		Gold standard: IC preferred
Calories	Initially: 50–65% of energy needs First 2 weeks: 25–30 kcal/kg/day	Permissive mild underfeeding:
		Short term
		With stabilization: meet energy needs
Protein	1.5–2.0 g/kg/day	Equilibrate nitrogen balance
		Decrease fat free mass catabolism (skeletal muscle)

*GI, gastrointestinal; GALT, gut-associated lymphoid system; IC, indirect calorimetry.*

Finally, all the macronutrients included in the whole process of nutrition therapy must be complemented with all the necessary micronutrients, such as vitamins, minerals, and trace elements (mainly zinc, copper, manganese, chromium, and selenium), respecting their daily required ingestion (DRI), for healthy and balanced nutrition in this critical clinical situation. Normally, all these micronutrients are included either in the enteral or parenteral formulations, at least in concentrations respecting their DRI. Their deficiencies or excesses should be regularly monitored and corrected on a daily basis in the routine process of nutrition therapy.

It is noteworthy to mention the specific roles of vitamins D and E, magnesium and zinc in the nutrition management of TBI patients. Vitamin D in the form of 1,25(OH)2-D3 and vitamin A in the form of 9-*cis-*retinoic acid act together in the CNS, where they seem to participate in cell proliferation and neuronal differentiation and function. Then, vitamin D in association with progesterone, which is also produced in the cerebral tissue and called neuroprogesterone, can subsequently influence neuronal excitotoxicity and apoptosis and may also promote myelin repair, exhibiting potential neuroprotective mechanisms in the context of TBI (Scrimgeour and Condlin, 2014).

Razmkon et al. (2011) randomized patients with severe head injury to receive vitamins C and E as opposed to controls on standard treatment, determining that patients in the treatment group had lower mortality rates and better Glasgow Outcome Scale (GOS) scores, but the sample size and methodological concerns in this study advise for further investigation (Razmkon et al., 2011).

Magnesium, which is transported to the brain by an active mechanism, in normal conditions inhibits the actions of the excitatory neurotransmitter glutamate, relaxes vascular smooth muscle, resulting in vasodilation and increased cerebral blood flow, and moreover, plays an important role in the homeostatic regulation of the pathways involved in the secondary phase of brain injury. Several human and animal studies that evaluated the effectiveness of magnesium administration in providing resilience or treating TBI in the acute phase showed favorable effects. However, they also show that the most critical issue yet to be addressed is the window of opportunity for magnesium use in the treatment of TBI, which probably could be up to 3 h after the onset of TBI. Recently, Nayak et al. (2018) studied 72 patients with sTBI, based on GCS and GOS scores, and demonstrated that 58% of patients had low serum magnesium levels (<1.3 mEq/L) at admission. At the 6-month follow-up, 81% of patients with poor neurological outcomes had low serum magnesium compared to 19% of patients with good outcomes (*p* = 0.01). Hypomagnesemia was associated with poor neurological outcome (odds ratio = 2.1, *p* = 0.04, 95% CI = 1.0–8.8) on regression analysis. In conclusion, the authors stated that hypomagnesemia appears to be an independent prognostic marker in patients with sTBI (Nayak et al., 2018).

On average, 10% of CNS zinc is in the free form and is associated with presynaptic vesicles of glutamatergic neurons. Free zinc also has important neuromodulatory roles. However, it has been shown that excessive release of zinc from synaptic boutons can result in post-synaptic neuronal death, and those neurons in brain regions with high concentrations of free zinc, such as the hippocampus, are thus especially prone to zinc-mediated damage and death. Nevertheless, TBI results in significantly depressed serum zinc levels as well as increased urinary zinc excretion, which is proportional to the severity of brain injury and can reach 14 times normal values, suggesting rapid zinc depletion in sTBI (Morris and Levenson, 2013). As TBI induces various damaging oxidative processes, several studies have shown a role for zinc deficiency in the induction of reactive oxygen species (ROS), thus exacerbating oxidative damage. Likewise, a low concentration of Zn in peripheral blood is associated with depression, and as many as 40% of hospitalized patients with TBI develop major depression, making it the most common long-term complication of TBI. Thus, experimental and clinical evidence aimed at a possible role of Zn as a treatment in TBI suggests that in the acute care situation following TBI, Zn deficiency should be prevented to maintain visceral protein stores and to optimize the potential for neurological recovery, which could increase resilience and improve brain injury outcomes. The only dose of supplemental Zn that has been tested in a clinical setting is 12 mg Zn-sulfate/day administered intravenously for the first 15 days after injury. After day 15, an oral dose of 22 mg/day was provided. Therefore, considering the scarcity of clinical trials, the existing data strongly suggest that Zn status should be monitored and maintained after a brain injury, not only because of the reported neurocognitive outcomes but also because both Zn deficiency and TBI have been associated with oxidative stress (Cope et al., 2012; Morris and Levenson, 2013).

## Brain Metabolism and Multimodal Monitoring

### The Role of Cerebral Microdialysis

**FIGURE 3:**
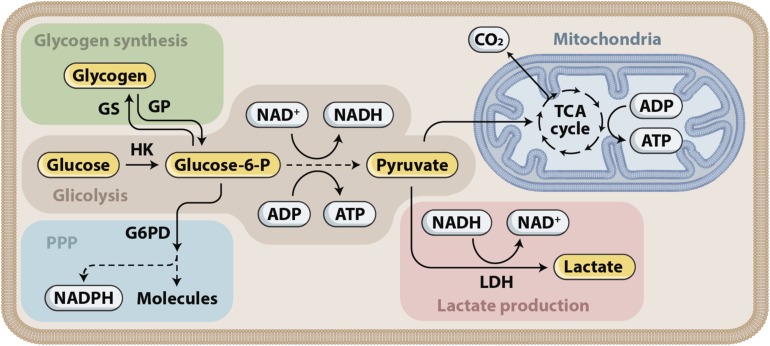
Glucose metabolism. Glucose is first phosphorylated to glucose-6-phosphate (glucose-6-P), which has three fates that correspond to the three main functions of glucose. First, energy can be stored as glycogen. Glycogen can later be mobilized and subsequently metabolized to pyruvate. Second, energy in the form of ATP can be produced by glucose-6-P entering glycolysis, supplying pyruvate for the tricarboxylic acid (TCA) cycle in the mitochondria and the associated oxidative phosphorylation. Glycolysis produces ATP and NADH. Depending on the cell type, pyruvate can also be converted into lactate through the action of lactate dehydrogenase (LDH). Third, reducing equivalents in the form of NADPH are produced in the pentose phosphate pathway (PPP).

**FIGURE 4:**
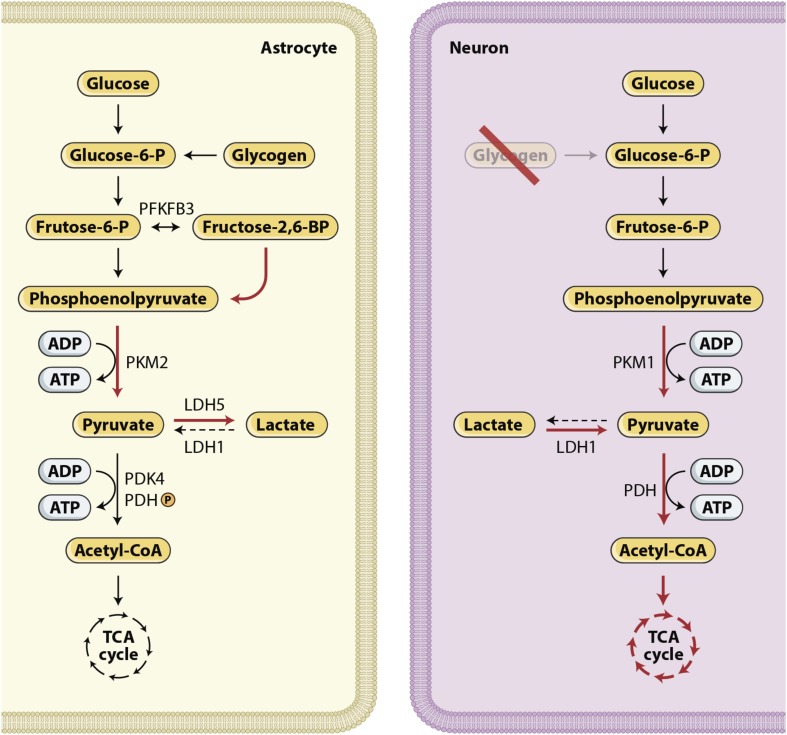
Glucose metabolism in the brain. The expression of different metabolic pathways for glucose is cell specific. Red arrows show upregulated pathways and black dashed arrows show downregulated pathways. Lactate production characterize astrocytes, whereas neurons use a significant proportion of glucose in the PPP (Bouzier-Sore and Bolanos, 2015) and use lactate, after its conversion to pyruvate, as their preferred mitochondrial energy substrate (Patet et al., 2016; Magistretti and Allaman, 2018). In summary, these cell-specific expression and activity profiles confer to neurons a restricted potential for upregulating glycolysis and an active oxidative phosphorylation activity. By contrast, in astrocytes, aerobic glycolysis is favored and oxidative activity is limited.

The injured brain develops a high demand for energy met by oxidative metabolism of oxygen and glucose. An inadequate supply of either of these substrates in patients who suffered severe TBI leads to neurological deterioration, secondary brain injury, and eventually death. Glucose is the main metabolic substrate used by the brain under normal conditions. During recent years, the astrocyte–neuron lactate shuttle (ANLS) hypothesis has emerged, stating that astrocytes produce lactate, which is then taken up by the adjacent neurons and used as an alternative energy substrate (Pellerin et al., 2007; Pellerin and Magistretti, 2012), as shown in Figures 3, 4. Cerebral microdialysis (CMD) is a unique tool that allows for real-time monitoring of brain energy metabolism through the measurement of specific metabolites’ concentrations in the brain tissue extracellular space.

Cerebral microdialysis is an invasive technique that consists of the insertion of a small probe approximately 2 cm deep into the brain parenchyma, usually in viable but at-risk regions. The CMD catheter has a semi-permeable dialysis membrane that allows measurement of the concentrations of metabolites in the brain tissue extracellular space. This semi-continuous bedside neurochemical evaluation reveals metabolic patterns that are associated with normal or altered brain homeostasis. Analysis of hourly measures of brain glucose, lactate, pyruvate, and glutamate permits a better understating of pathophysiological processes leading to imbalances in supply and demand. Early recognition of these patterns and imbalances, especially when analyzed in combination with other multimodal monitoring parameters such as intracranial pressure (ICP), brain tissue oxygen (PtiO2) and electroencephalography (EEG), may create a window of opportunity for clinical interventions that prevent definite secondary brain injury (Stocchetti et al., 2013; Citerio et al., 2015; Hutchinson et al., 2015; Oddo and Hutchinson, 2018).

A number of studies have demonstrated the utility of CMD both as a clinical research tool and as part of cerebral multimodal monitoring of patients with severe TBI. The development of certain MD patterns, such as increased lactate/pyruvate ratio (LPR) and low glucose, have been shown to precede common complications of TBI patients, such as ischemia, seizures and intracranial hypertension (Vespa, 2006; Vespa et al., 2007; Stocchetti et al., 2013). These metabolic distress patterns usually reflect imbalances in substrate supply and energy demand, which allows for the individualization of therapy before irreversible injury ensues. Consistent observational data are also available, linking altered MD parameters to worse functional outcomes and mortality after TBI (Vespa et al., 2003, 2005; Vespa, 2006; Timofeev et al., 2011). This observation has led to the early identification of patients at higher risk that deserve more aggressive treatment. Furthermore, CMD has allowed the improvement of our understanding of novel mechanisms involved in brain metabolism and future treatment approaches (Quintard et al., 2016; Patet et al., 2016; Carteron et al., 2017, 2018; Bernini et al., 2018; Oddo and Hutchinson, 2018).

### Systemic and Brain Glucose

Dysglycemia is a common complication in patients with TBI. As previously mentioned, TBI generates a systemic hypermetabolic state associated with hyperglycemia. The presence and severity of hyperglycemia have been repeatedly been shown in the literature to correspond to the severity of injury and poor clinical outcomes after TBI. At the brain level, the usual consequences of neurotrauma related to glucose metabolism include hyperglycolysis, mitochondrial dysfunction, and low or high CMD glucose (Vespa et al., 2003; O’Connell et al., 2005; Hutchinson et al., 2009; Timofeev et al., 2011; Kurtz et al., 2013; Jalloh et al., 2015a, b, 2018). Other than neurotrauma itself, medical interventions in the ICU may affect glucose metabolism, such as enteral/parenteral nutrition and insulin therapy (Vespa et al., 2006, 2012; Schmidt et al., 2012; Badjatia and Vespa, 2014; Kofler et al., 2018). Excess exogenous glucose or total carbohydrate administration, exceeding the patient’s glucose oxidation rate (maximum of 5 mg/kg/min, or 0.3 g/kg/h), may lead to hyperglycemia. Thus, hyperglycemia probably reflects the intensity of the stress response and the severity of the primary injury and may be worsened by clinical management in the ICU (Donnelly et al., 2015).

Abundant data confirm that hyperglycemia is deleterious after TBI (Rostami, 2014; Hermanides et al., 2018). However, the threshold for aggressive glucose control treatment remains a matter of debate. Following two large single-center studies by van Den Berghe and associates in the early 2000s (van den Berghe et al., 2001; Van den Berghe et al., 2006), suggesting that tight glycemic control may benefit general critically ill patients, this strategy was widely implemented. At that time, researchers raised concerns that the beneficial effect of aggressive insulin therapy might have been related to a disproportionate use of parenteral nutrition and iatrogenic hypoglycemia. However, it was not until the publication of the multicenter study NICE-SUGAR, which showed no benefit of intensive vs. moderate glucose control in the general ICU and the *post hoc* analysis of its neurotrauma subgroup demonstrating more frequent episodes of hypoglycemia, that a more liberal glucose control strategy was accepted and recommended for patients with TBI (Investigators et al., 2009; Nice-Sugar Study Investigators for the Australian, New Zealand Intensive Care Society Clinical Trials Group, and the Canadian Critical Care Trials Group et al., 2015; Carney et al., 2017). To add to the controversy, a recent systematic review of 10 trials comparing conventional vs. intensive glycemic control found no effect on mortality but a borderline increased risk of poor neurological outcome in the conventional group (Hermanides et al., 2018).

While the controversy around intensive glucose control with insulin still remains, some research groups have focused their attention on its effects on brain energy metabolism as measured by CMD. Vespa and colleagues examined the effects of glycemic control in 47 moderate-to-severe TBI patients, 33 with loose insulin and 14 with intensive insulin therapy (glycemic target 80 to 110 mg/dL). There was a reduction in MD glucose of 70% from the baseline concentration in the intensive therapy group compared to a 15% reduction in the patients treated with the loose insulin protocol. Despite this reduction in glucose availability, the global metabolic rate of glucose did not change. However, intensive insulin therapy was associated with markers of neurotoxicity and metabolic distress, such as higher glutamate (*p* < 0.01) and LPR (*p* < 0.03) and lower brain glucose (*p* < 0.05) (Vespa et al., 2006). Similar findings from observational and comparative studies later confirmed the increased risk of brain metabolic distress and reduced brain glucose related to aggressive insulin treatment and tight glycemic control (Vespa et al., 2006; Oddo et al., 2008; Schlenk et al., 2008; Meierhans et al., 2010; Plummer et al., 2018).

Other than the impact of intensive insulin therapy on the brain, CMD studies have investigated other potential relationships between systemic glucose and brain metabolism after TBI. Mild and moderate hypoglycemia have been associated with low brain glucose and increased LPR, mainly caused by lower pyruvate levels (Magnoni et al., 2012; Kurtz et al., 2013). The mechanisms that explain these results are still poorly understood but may involve impaired glucose transport to the brain and increased glucose utilization. These findings highlight the importance of adequate substrate availability to maintain oxidative metabolism in conditions of high demand and stress the link between adequate nutrition therapy after TBI and preservation of brain function. Based on this concept, recent guidelines have confirmed the clinical usefulness of CMD to detect reduced availability of brain glucose and to individualize glycemic targets to avoid metabolic failure (Hutchinson et al., 2015).

### Brain Metabolic Patterns and Optimization of Substrate Supply

The main goal of neurocritical care of severe TBI patients is to avoid secondary insults to an injured and vulnerable brain. CMD is widely used to promote early detection of ischemia and metabolic crisis after TBI. The most commonly described pathologic CMD patterns in patients with severe TBI are increased LPR and reduced brain glucose. Elevated LPR has consistently been associated with increased mortality and unfavorable outcomes after TBI (Goodman et al., 1999; Timofeev et al., 2011). While reduced brain glucose has been shown to predict poor outcome in various studies, analysis of a large cohort of TBI patients monitored with MD demonstrated in a multivariable model that higher brain glucose was, in fact, a predictor of mortality (Vespa et al., 2003; Timofeev et al., 2011; Kurtz et al., 2013; Citerio et al., 2015). This observation is likely due to the low incidence of hypoglycemia and cerebral ischemia in that cohort, minimizing the frequency of episodes and potential impact of reduced cerebral glucose on outcomes. On the other hand, higher cerebral glucose is likely to reflect hyperglycemia, which has a well-known deleterious effect on patients with neurological injury. The outcome data available suggest that elevated LPR (> 25 or > 40, depending on the study) as well as reduced (< 1 mmol/L) and elevated brain glucose are associated with worse outcomes after TBI.

The LPR reflects the brain tissue metabolic state, and its elevation may reflect the presence of either mitochondrial dysfunction (Verweij et al., 2000) or lack of oxygen supply due to ischemia or hypoxia (Hlatky et al., 2004). In the case of ischemia, cerebral blood flow is impaired, brain tissue oxygen is reduced (PtiO2 < 15 mmHg) and aerobic metabolism is compromised. However, after the hyper-acute phase, data from PET studies have shown that ischemia is not as frequent as previously thought in TBI (Vespa et al., 2005; Hutchinson et al., 2009). In the absence of ischemia, metabolic crisis may be predominantly attributable to increased glycolysis or mitochondrial dysfunction (impairment of oxygen utilization or cytopathic hypoxia) (Vespa et al., 2005; Sala et al., 2013). In this context, pyruvate may be normal or elevated, and elevations of CMD lactate and LPR are lower than during frank ischemia/hypoxia. Indeed, recent evidence from CT perfusion and CMD studies confirm that most of the extracellular lactate increase (>4 mmol/L) in TBI is predominantly non-ischemic and is mainly related to activated glycolysis (Sala et al., 2013; Bouzat and Oddo, 2014; Bouzat et al., 2014).

Reduced cerebral glucose may be the end result of different pathophysiological processes. The classical CMD description of metabolic crisis due to ischemia includes low cerebral glucose (<0.7 mmol/L) (Frykholm et al., 2005). More recently, CMD low glucose (<1 mmol/L) was independently associated with decreased CBF (<35 mL/100 g/min) and, combined with ICP and PtiO2, improved the accuracy of detection of hypoperfusion in TBI as measured by perfusion CT (Bouzat et al., 2015).

As previously described, low brain glucose can also reflect moderate-to-severe reductions in systemic glucose or impaired glucose uptake or utilization by the brain in normal or mildly reduced glycemia (Magnoni et al., 2012; Kurtz et al., 2013). In situations of preserved regional perfusion and glycemia, the mechanism related to low CMD glucose is the same one that leads to elevated lactate, namely, oxidative glycolysis. Clinical investigations using PET scans and CMD have shown that TBI is associated with increased cerebral glucose utilization that may eventually culminate in a state of energy dysfunction (Bergsneider et al., 1997; Glenn et al., 2003). In this setting, the supply of glucose to the injured brain, the main energy substrate, may become limited, leading to reductions in cerebral extracellular glucose below critical thresholds. Emerging evidence has demonstrated that the brain can use substrates other than glucose to sustain increased activity, including lactate (Bouzat et al., 2014; Quintard et al., 2016; Patet et al., 2016; Carteron et al., 2018; Magistretti and Allaman, 2018).

As the main energy substrate of the brain, the glucose supply should be optimized to preserve brain function. To achieve this goal, a comprehensive understanding of the interplay between nutrition therapy, glycemic control, insulin therapy and brain metabolic profiles, as measured by bedside CMD, is needed. There is little evidence of the effects of nutrition therapy on the CMD parameters of TBI and neurocritical patients in general. Recently, reports on comatose patients with subarachnoid hemorrhage have shown conflicting results. Schmidt et al. (2012) failed to demonstrate an association between nutrition therapy and improvement of brain glucose. In parallel, they showed that insulin was associated with reductions in brain glucose independent of serum glucose (Schmidt et al., 2012). On the other hand, Kofler et al. (2018) recently found significant increases in CMD-glucose levels, independent of the baseline brain metabolic profile and insulin administration. Moreover, significant changes in CMD glucose were observed in both pericontusional and normal-appearing tissue (Kofler et al., 2018).

Although there are no such data on TBI patients, we can extrapolate that optimal nutrition and substrate supply may potentially have beneficial effects on the brain metabolic profile. Integrating these concepts into current guideline recommendations on nutrition therapy and the use of CMD after severe TBI from the Brain Trauma Foundation and the International Multi-disciplinary Consensus Conference on Multimodality Monitoring (Le Roux et al., 2014; Carney et al., 2017), we suggest the implementation of early EN (within 24 to 48 h) with at least 50% of calculated or measured energy needs, followed by a more aggressive progression to full caloric replacement depending on the presence of reduced cerebral glucose and elevated LPR. Concomitantly, clinicians should avoid hyperglycemia (>180 mg/dL) and implement insulin therapy carefully to prevent hypoglycemia (Table 4).

**TABLE 4:** Guideline and consensus statement recommendations on nutrition therapy, glucose control, and cerebral metabolism in traumatic brain injury patients.

Topic	Subtopic	Recommendations and level/strength	Comments by the authors	References
Nutrition therapy	Timing of feeding after injury	“Feeding patients to attain Resting Energy Expenditure (REE) requirements at least by the fifth day and, at most, by the seventh day post-injury is recommended to decrease mortality (Level IIA)”	The REE should always ideally be measured by Indirect Calorimetry (IC), if possible. If not, administer 25 kcal/kg/day, or 70% of the measured or estimated REE, during the initial 7 to 10 days. The authors also suggest EN initiation preferably, as soon as the patient is fully resuscitated and tolerant, within 24 to 48 h of injury.	Brain Trauma Foundation Guidelines 2016 (Carney et al., 2017)
		“Early EN (<48 h) should be performed in patients with traumatic brain injury (Grade of recommendation: B – strong consensus [95.83% agreement])”		ESPEN 2019 (Singer et al., 2019)
		“We recommend that, similar to other critically ill patients, early enteral feeding be initiated in the immediate post-trauma period (within 24–48 h of injury) once the patient is hemodynamically stable.” (quality of evidence: very low)”		SCCM-ASPEN 2016 Guidelines (McClave et al., 2016)
	Method of feeding	“Transgastric jejunal feeding is recommended to reduce the incidence of ventilator-associated pneumonia (Level IIB)”	Intragastric enteral nutrition is usually physiologically better, enhances GI and Systemic Immunities, as long as the patient tolerates it. If not, the following options are transgastric jejunal feeding, or PN.	Brain Trauma Foundation Guidelines 2016 (Carney et al., 2017)
		“Trauma patients should preferentially receive early EN instead of early PN.” (Grade of recommendation: B – strong consensus [96% agreement])		ESPEN 2019 (Singer et al., 2019)
	Vitamins and supplements	“There is insufficient evidence about the influence of vitamins and supplements to inform recommendations.”	Vitamins and trace elements, of which mainly selenium, presumably have enhanced metabolic consumption in the acute phase of TBI, but without scientific confirmation. The immune-modulating formulations are markedly anti-inflammatory and stimulate protein synthesis.	Brain Trauma Foundation Guidelines 2016 (Carney et al., 2017)
		“Based on expert consensus, we suggest the use of either arginine-containing immune-modulating formulations or EPA/DHA supplement with standard enteral formula in patients with TBI.”		SCCM-ASPEN 2016 Guidelines (McClave et al., 2016)
	Monitoring	“We recommend against routine monitoring of gastric residuals in mechanically ventilated patients (strong recommendation, high quality of evidence).”	The authors agree with this recommendation. The presence of elevated gastric residuals will not enhance the risk of its pulmonary aspiration but indicate enteral nutrition intolerance and consequently underfeeding.	Consensus Statement – NCS and ESICM 2014 (Le Roux et al., 2014)
	Monitoring	“We suggest against the routine monitoring of nutritional requirements with measurement of energy expenditure by indirect calorimetry or the use of predictive equations for assessing nutritional requirements (weak recommendation, low quality of evidence).”	Almost routinely patients with TBI are under sedation and neuromuscular blockade, which will result on an altered value for the measured REE. However, IC is still the main recommendation by the Nutrition Societies for measuring the actual REE in critically ill patients.	Consensus Statement – NCS and ESICM 2014 (Le Roux et al., 2014)
	Monitoring	“In critically ill mechanically ventilated patients, Energy Expenditure (EE) should be determined by using IC – Grade recommendation: B and strong consensus (95% agreement); if IC is not available, using VO2 (oxygen consumption) from pulmonary arterial catheter or VCO2 (carbon dioxide production) derived from the ventilator will give a better evaluation on EE than predictive equations. Consensus (82% agreement)”	REE (as measured by VCO2 × 8.19) has been demonstrated to be more accurate than predictive equations (Stapel et al., 2015)	ESPEN 2019 (Singer et al., 2019)
	Monitoring	“We recognize that accurately measuring nitrogen balance is difficult, but where this is possible, we suggest that this may be used to help assess the adequacy of nutritional support (weak recommendation, very low quality of evidence).”	If the patients do not have an intestinal or extra-intestinal nitrogen loss, the nitrogen balance is usually accurate enough, and will greatly help in modulating protein catabolism and its adequate supply.	Consensus Statement – NCS and ESICM 2014 (Le Roux et al., 2014)
Glucose control	Aggressive vs. conventional targets	“Given the lack of consistency in these findings, it is not clear whether aggressive therapy is better than conventional glucose control. For this reason, the evidence was rated as insufficient and no recommendation about glucose control can be made at this time.”	Based on the evidence that hypoglycemia is more common during aggressive glucose control and that hyperglycemia is associated with worse outcomes, we suggest avoiding hypoglycemia (< 80–100 mg/dL) and hyperglycemia (> 180 mg/dL)	Brain Trauma Foundation Guidelines 2016 (Carney et al., 2017)
	Avoiding hypoglycemia	“We recommend that arterial or venous blood glucose be measured by a laboratory-quality glucose measurement immediately upon admission, to confirm hypoglycemia, and during low perfusion states for patients with acute brain injury” (Strong recommendation, High quality of evidence).	It is crucial to avoid or rapidly correct severe hypoglycemia (< 40 mg/dL)	Consensus Statement – NCS and ESICM 2014 (Le Roux et al., 2014)
	Monitoring	“We recommend serial blood glucose measurements using point-of-care testing should be performed routinely during critical care after acute brain injury.” (Strong recommendation, High quality of evidence).	We monitor patients with severe TBI every 1 to 2 h in the first 7 days after trauma	Consensus Statement – NCS and ESICM 2014 (Le Roux et al., 2014)
Brain Metabolism	Cerebral microdialysis (CMD)	“We suggest the use of CMD to assist titration of medical therapies such as systemic glucose control and the treatment of delayed cerebral ischemia” (weak recommendation, moderate quality of evidence).	CMD may be used to help guide systemic glycemic control, specifically to avoid cerebral hypoglycorrhachia in comatose TBI patients	Consensus Statement – NCS and ESICM 2014 (Le Roux et al., 2014)
	Cerebral microdialysis (CMD)	“We recommend monitoring CMD in patients with or at risk of cerebral ischemia, hypoxia, energy failure, and glucose deprivation” (strong recommendation, low quality of evidence).	Increased Lactate/Pyruvate Ratios, as well as low Glucose may indicate cerebral metabolic distress.	Consensus Statement – NCS and ESICM 2014 (Le Roux et al., 2014)
	Cerebral microdialysis for neuroprognostication	“While persistently low brain glucose and/or an elevated lactate/pyruvate ratio is a strong predictor of mortality and unfavorable outcome, we recommend that cerebral microdialysis only be used in combination with clinical indicators and other monitoring modalities for prognostication” (strong recommendation, low quality of evidence).	CMD parameters should never be used in isolation to prognosticate after TBI.	Consensus Statement – NCS and ESICM 2014 (Le Roux et al., 2014)

*EN, enteral nutrition; PN, parenteral nutrition; TBI, traumatic brain injury; CMD, cerebral microdialysis; NCS, Neurocritical Care Society; ESICM, European Society of Intensive Care Medicine; SCCM, Society of Critical Care Medicine.*

### New Insights on Neuroenergetics and Energy Fuel Supply

Mounting evidence in recent years has suggested that lactate is used as an alternative fuel to maintain homeostasis in brain injured patients (Sala et al., 2013; Patet et al., 2016; Magistretti and Allaman, 2018). Lactate is formed both through anaerobic metabolism and through glycolysis. The glycolytic pathway is one of the various metabolic ways through which glucose can be processed to produce energy. With normal oxygen tension, ATP is mostly produced through the mitochondrial electron transport chain. Glucose is processed to pyruvate, which enters the tricarboxylic acid (TCA) cycle and promotes the most efficient energy pathway, resulting in the production of 32–36 ATP molecules. By contrast, under hypoxic or anoxic conditions, pyruvate is converted to lactate through anaerobic metabolism, generating only 2 ATP molecules per glucose molecule. Another way to process glucose is aerobic glycolysis. In aerobic glycolysis, lactate is formed despite the presence of normal oxygen tension. This metabolic processing of glucose is typical of astrocytes and is due to a cell specific gene expression profile that favors the conversion of pyruvate to lactate rather than the use of pyruvate in the TCA cycle. The relevance of the so-called astrocyte-neuron lactate shuttle (ANLS), described decades ago (Pellerin and Magistretti, 2012), in patients with severe acute brain injury has been increasingly revealed by clinical research over the last few years (Figure 4).

Clinical investigation assessing brain metabolism with positron emission tomography, magnetic resonance spectroscopy and CMD has repeatedly disclosed major alterations of cerebral energy metabolism in the aftermath of TBI. These studies have revealed that metabolic impairment is characterized by increased glycolysis, high glucose demand and diversion of glucose to reparative pathways, such as the pentose phosphate pathway (Jalloh et al., 2015b). Ultimately, these processes lead to reduced glucose availability. To compensate for this glucose shortage, lactate metabolism and uptake are increased. Some authors argue that this use of alternative cerebral energy substrates, including lactate and ketone bodies, may be an adaptive mechanism following TBI (Oddo et al., 2019).

Lactate has repeatedly been shown to function as an alternative fuel for the injured brain. As mentioned, before aerobic glycolysis takes place in the astrocyte without oxidative metabolism of substrates. Lactate is then transferred to neurons, providing additional energy substrate while also acting as a signaling molecule for other processes. This knowledge has led not only to a better understanding of the cerebral metabolic impairment in TBI but also to therapeutic options that offer lactate supplementation to ameliorate brain dysfunction (Bouzat et al., 2014; Quintard et al., 2016; Carteron et al., 2018). The administration of hypertonic lactate solutions demonstrated beneficial effects in patients with TBI. Reduced ICP and MD glutamate, increased MD glucose and improved CBF are among the potential benefits of this treatment option. These authors suggest that the glucose-sparing effects of lactate supplementation and the increased glucose availability that results are the main drivers of the metabolic improvements observed. On the other hand, critics argue that CMD metabolites’ concentrations alone do not reflect metabolic processes and that the effects observed after hypertonic lactate administration may be the result of ICP reduction and improvements in cerebral perfusion. Furthermore, concerns have been raised that lactate “flooding” of the brain may not only simulate metabolic profile improvements but also be deleterious to brain energy production (Nordstrom and Nielsen, 2014; Dienel et al., 2016). To date, there are no studies demonstrating improvements in clinically important outcomes of lactate supplementation following TBI. Thus, the use of hypertonic lactate to improve brain energy metabolism remains investigational. However, some authors argue that, because of its safety and effectiveness, hypertonic lactate could replace hypertonic saline and mannitol as the preferred therapeutic option of hyperosmolar therapy in patients with TBI that develop intracranial hypertension (Bouzat et al., 2014; Carteron et al., 2017).

Other potential fuel supplementations for TBI patients are succinate and ketone therapy. In small elegantly conducted human studies, locally administered (through CMD perfusion) succinate, a tricarboxylic acid cycle intermediate interacting directly with the mitochondrial electron transport chain, improved brain chemistry and improved energy metabolism in TBI patients with evidence of cerebral mitochondrial dysfunction (Jalloh et al., 2017; Stovell et al., 2018). With more practical implications, exogenous ketone can be delivered in the form of ketogenic diets, using enteral formulae enriched with medium-chain triglycerides and containing low doses of carbohydrates. Ketogenic diets have been tested successfully in neurocritical care patients with refractory epilepsy. Ketone supplementation or ketogenic diets have various neuroprotective effects in experimental and clinical models of brain injury, acting on seizure control and oxidative stress (Greco et al., 2016; McDougall et al., 2018). Recent clinical studies have shown that fasting promotes increases in ketone body concentration in CMD—suggesting effective transfer from the systemic circulation and a potential therapeutic role in TBI—and that intravenous ketone body administration improved cerebral metabolism by a glucose-sparing effect and increases in cerebral blood flow (Bernini et al., 2018; Svart et al., 2018).

## Conclusion

Nutrition therapy is a cornerstone in the management of patients suffering a severe TBI. Nutrition strategies and monitoring affect systemic glucose control and consequently, brain metabolic function. An integrated approach that includes early and optimized nutrition, moderately aggressive glucose control, and multimodal monitoring using CMD to avoid neuroglycopenia and metabolic distress, is probably the best option to avoid secondary brain injury and to improve outcomes. Exciting new data on substrate supplementation for brain energy production may change the way we feed patients with severe brain injury in the future. In the meantime, we need to improve our bedside monitoring tools and better understand the cerebral metabolic effects of therapeutic interventions. Finally, safe and effective treatments should demonstrate improvements in clinically important outcomes such as mortality and long-term cognition.

## Author Contributions

All authors listed have made a substantial, direct and intellectual contribution to the work, and approved it for publication.

## Conflict of Interest

The authors declare that the research was conducted in the absence of any commercial or financial relationships that could be construed as a potential conflict of interest.
